# Neurochemical and Behavioral Characteristics of Toxic Milk Mice: An Animal Model of Wilson’s Disease

**DOI:** 10.1007/s11064-013-1111-3

**Published:** 2013-07-23

**Authors:** Adam Przybyłkowski, Grażyna Gromadzka, Adriana Wawer, Ewa Bulska, Katarzyna Jabłonka-Salach, Tomasz Grygorowicz, Anna Schnejder-Pachołek, Andrzej Członkowski

**Affiliations:** 1 Department of Clinical and Experimental Pharmacology, Medical University of Warsaw, ul. Krakowskie Przedmiescie 26/28, 00-927 Warsaw, Poland; 2 Department of Gastroenterology, Endoterapia, Warsaw, Poland; 3 2nd Department of Neurology, Institute of Psychiatry and Neurology, Warsaw, Poland; 4 Laboratory of Basic Aspects of Analytical Chemistry, Faculty of Chemistry, University of Warsaw, Warsaw, Poland

**Keywords:** Toxic milk mice, Wilson’s disease, Neuropathology

## Abstract

Toxic milk mice have an inherited defect of copper metabolism. Hepatic phenotype of the toxic milk mice is similar to clinical findings in humans suffering from Wilson’s disease (WND). In the present study, neurotransmitter system and locomotor performance in toxic milk mice was examined to verify the feasibility of this animal model for studying neuropathology of WND. Mice aged 2 and 12 months were used in the experiment. The mice were tested according to rotarod and footprint protocols. Monoamine content in brain structures was measured by high performance liquid chromatography. In order to detect neuronal loss, expression of enzymes specific for dopaminergic [tyrosine hydroxylase (TH)], noradrenergic (dopamine beta-hydroxylase) and serotoninergic [tryptophan hydroxylase (TPH)] neurons was analyzed by Western blot. The 12-month-old toxic milk mice demonstrated impaired locomotor performance in behavioral tests. Motor deficits were accompanied by increased copper and serotonin content in different brain regions and slight decrease in dopamine concentration in the striatum. The expression of TH, dopamine beta-hydroxylase and TPH in the various brain structures did not differ between toxic milk mice and control animals. Despite differences in brain pathology between humans and rodents, further exploration of neuronal injury in toxic milk mice is warranted to broaden the understanding of neuropathology in WND.

## Introduction

Wilson’s disease (WND) is a genetic disorder of copper metabolism [1]. The affected gene is localized on chromosome 13 in humans and codes for ATP7B protein, a copper-transporting P-type adenosine triphosphatase (ATPase). Dysfunction of ATP7B triggers accumulation of copper within liver, brain and other organs. Excessive copper levels cause organ damage which is responsible for occurrence of wide spectrum of hepatic and neuropsychiatric symptoms. Hereditary copper metabolism disorders have also been discovered in animals. Mutations in rodent orthologue of *ATP7B* lead to hepatic phenotype similar to WND in Long Evans Cinnamon (LEC) rats and in toxic milk mice [2]. Toxic milk (*tx*) is an autosomal recessive mutation in *Atp7b* gene in C57BL/6J mice strain. This mutation arose spontaneously in laboratory of University of Massachusetts (MA, USA) and was characterized by Rauch (thus this strain will be referred in the text as *txR*) [3]. Another recessive mutation in murine *Atp7b* gene was discovered in C3H/HeJ strain in The Jackson Laboratory (ME, USA). The toxic milk mutation detected in The Jackson Laboratory (*txJ*) is a point mutation at position 2135 (in exon 8) which leads to glycine to aspartic acid (G712D) missense mutation in ATPase [4]. This specific mutation has not been reported in any human WND patient; however, the *txJ* mice share phenotypic similarities with WND. In *txJ* mice, copper accumulates in liver, brain and other organs [5–7]. Both in patients with WND and in toxic milk mice it was observed that malfunction of ATP7B results in incomplete synthesis of cuproenzyme–ceruloplasmin [8]. Apoceruloplasmin is quickly degraded thus the serum ceruloplasmin concentration is low in affected humans and animals [7, 8]. Involvement of liver in toxic milk mice characterized by steatosis, mild inflammation and gross nodularity resembles pathology observed in humans affected by WND [1, 5, 6].

Neurological signs are present in 40 % of patients with WND [8]. The typical brain pathology observed in WND is bilateral degeneration of the putamen and globus pallidus, the other changes include lesions in the caudate, thalamus, brainstem and cerebellum [9]. The concentration of metabolites of noradrenaline, serotonin and dopamine in cerebrospinal fluid of WND individuals is decreased which reflects damage of different neuronal population [10, 11]. The neurological symptoms observed in WND are classified in different syndromes such as akinetic-rigid syndrome similar to Parkinson’s disease, pseudosclerosis with tremor as dominating symptom, ataxia, and dystonic syndrome [1].

There are only a few reports of neuronal injury and neurological symptoms in The Jackson Laboratory strain of toxic milk mice [7, 12]. In order to establish whether brain involvement in *txJ* animal model can be used for WND neuropathology studies, neurochemical and behavioral phenotypes were examined in young and aged animals. The neurological abnormalities observed in WND patients result from impaired motor coordination and gait disturbances. Therefore, *txJ* mice were tested by using two common rotarod protocols to assess locomotor coordination, while footprint test was chosen for the appraisal of locomotor gait. For detection of neuronal injury, neurotransmitters concentration, in different brain regions, as well as expression of enzymes specific for dopaminergic, noradrednergic and serotoninergic neurons was analyzed.

## Materials and Methods

All procedures were conducted in accordance with the European Communities Council Directive from the 24th November 1986 (86/609/EEC) and the Republic of Poland Animal (Scientific Procedures) Act. C3HeB/FeJ^Atp7btx−J/J^ and control C3HeB/FeJ mice were purchased from The Jackson Laboratory (Bar Harbor, ME, USA) and bred locally.

Pups of *tx* mice require fostering in order to survive thus were foster nursed within 5 days of birth to BALB/cByJ dams that produced litters at the similar time period. BALB/cByJ mice are recommended by The Jackson Laboratory for cross-fostering, as they are characterized by good reproductive performance and minimal aggression. After weaning, mice were housed within a 12/12 h inverted light/dark cycle in a temperature- and humidity-controlled environment. Same-sex and same-litter mice were housed 1–5/cage. Food and water (provided with lowered elongated spouts bottles) were available ad libitum. The experiments were performed on mice at both young (2 months old) and old (12 months old) age. Experimental groups comprised 8–10 animals (female–male 1:1). Tail biopsy was obtained from each mouse to check for *Atp7b* mutation and a unique number was tattooed on the remaining part of the tail for the purpose of identification. Thereafter, mice were trained and tested according to rotarod and footprint protocols. On the last day of behavioral testing, mice were sacrificed by cervical dislocation. Blood was collected for ceruloplasmin measurement. Upon removal of the brain, striatum, hippocampus, frontal cerebral cortex and cerebellum were dissected and immediately frozen for further analysis.

### Ceruloplasmin Concentration

Serum ceruloplasmin oxidase activity in mice sera was analyzed using a modified method of Ravin [13]. Human serum was used as a positive control for the assay.

### Measurement of Metal Content

The brain samples were immersed in 1.5 ml of 65 % nitric acid (Merck, Darmstadt, Germany) for 48 h at room temperature, followed by addition of distilled water up to 5 ml (Milipore, MA, USA). Thereafter, all the aliquots were filtered using syringe polyamide-nylon filters. The accuracy of the analytical results was validated with the use of Rat Brain Reference Material with a certified content of copper. ^115^In was used as an internal standard. An Elan 6100 Dynamic Reaction Cell inductively coupled plasma mass spectrometer (ICP-MS; PerkinElmer, Waltham, USA) was used as element-specific detector, equipped with a Mainhard spray nebulizer, quartz Scott’s chamber and platinum cones. Under optimized conditions, the limit of detection was 10.9, 2.5 and 1.1 μg/kg for zinc, copper and iron, respectively.

### Measurement of Monoamines Content

Monoamines and corresponding metabolites concentrations were measured using high-performance liquid chromatography (HPLC) with electrochemical detection and glassy carbon electrode. Samples were homogenized in ice-cold 0.1 N HClO_4_ solution and centrifuged at 13,000×*g* for 15 min to precipitate proteins. Supernatant was removed, filtered using a filter paper of 0.20-μm pore size (Whatman, Kent, UK) and examined for contents of dopamine (DA), 3,4-dihydroxyphenylacetic acid (DOPAC), homovanillic acid (HVA), 3-methyltyrosine (3MT), noradrenaline (NA), 3-methoxy-4-hydroxyphenylglycol (MHPG), 5-hydroxytryptamine (5-HT), and 5-hydroxy-indoloacetic acid (5-HIAA) (standard substances supplied by Sigma-Aldrich, Poznan, Poland). An electrochemical potential was set at 0.8 V with respect to Ag/AgCl reference electrode. The mobile phase consisted of 31 mM sodium phosphate, 58 nM citric acid, 1 mM octanesulfonic acid, 27 mM ethylenediaminetetraacetic acid (EDTA) (Sigma-Aldrich, Poznan, Poland) in deionized (18.3 mV) polished water, 12 % acetonitrile and 1 % methanol (Merck, Darmstadt, Germany). Separation of monoamines was achieved with Nucleosil C-18 column, 250 × 4 mm, 5 μm particle size (Macherey–Nagel, Düren, Germany) with the mobile phase flow rate maintained at 0.8 ml/min. Samples were quantified by comparing with standard solutions of known concentration (external calibration) using ClarityChrom software (Knauer, Berlin, Germany).

### Measurement of Tyrosine Hydroxylase, Dopamine Beta-Hydroxylase and Tryptophan Hydroxylase Expression

In order to detect neuronal loss, expression of enzymes specific for serotoninergic, noradrenergic and dopaminergic neurons were analyzed by Western blot. Animals were sacrificed by decapitation. Brains were homogenized in radio-immunoprecipitation assay (RIPA) lysis buffer containing 50 mM Tris pH 7.4, 150 mM NaCl, 2 mM EDTA, 1 % NP-40, 0.25 % Na-deoxycholate, 1 mM phenylmethylsulfonyl fluoride (PMSF), and 1 mM Na_2_VO_3_ in the presence of protease inhibitor cocktail P8340 (all reagents supplied by Sigma-Aldrich, Poznan, Poland). Samples were incubated on ice for 40 min, then centrifuged at 10,000×*g* for 15 min. Supernatants were collected, and protein concentrations were determined via the Bradford method [14]. Samples containing 50 μg of proteins were subjected to SDS–polyacrylamide gel (10 %) electrophoresis and examined towards tyrosine hydroxylase (TH), tryptophan hydroxylase (TPH) and dopamine beta hydroxylase (DBH) protein. Samples were transferred to nitrocellulose membranes and incubated overnight (4 °C) with goat TH, DBH or TPH (Santa Cruz Biotechnology, TX, USA) primary polyclonal antibody in 1:200 dilution in 5 % nonfat milk in Tris-buffered saline with Tween 20 (TBST). Thereafter, membranes were incubated with secondary antibody conjugated with horseradish peroxidase (HRP) (Sigma-Aldrich, Poznan, Poland) in dilution 1:5,000. Monoclonal anti-actin antibody Santa Cruz Biotechnology, TX, USA) was used as a loading control. Bands were visualized with chemiluminescence ECL kit (Amersham/GE Healthcare, Freiburg, Germany) exposed to Hyperfilm ECL and quantified using densitometry Image Scanner III (Amersham/GE Healthcare, Freiburg, Germany) and with ImageJ 1.46 software (http://rsbweb.nih.gov/ij/). Relative protein expression was calculated by dividing the optical density of the protein of interest by the optical density of standard sample.

### Behavioral Tests

Behavioral tests were performed on mice maintained on an inverted day–light cycle in the dim light in the middle of the dark phase. Each animal’s weight was recorded on the first day of behavioral testing.

#### Rotarod

Mice aged 2 and 12 months were trained on the rotarod apparatus (Ugo Basile, Varese, Italy) for 3 consecutive days in an accelerated mode. Each mouse received 3 trials/day. The rotating speed increased from 5 to 40 rpm within 292 s, the cut-off was set at 300 s (5 min). The latency to fall off the rotarod for each mouse was recorded and used to generate group means. The same mice groups were tested on fixed-speed rotarod after 7 days of rest. The task consisted of giving each mouse three successive trials at 7 different speeds (ranging from 4 to 40 rpm). Inter-trial intervals were 10 min. At each speed, the latency to fall off the rotarod (the average of the 3 trials) was used. The maximum latency was 60 s.

#### Footprint Test

Footprint test was performed with a 12-cm wide, 60-cm long runway (with 13-cm high walls). Before testing, mice were allowed to explore the runway for 5–10 min. Subsequently, the mice completed 3–5 trial runs. Footprint recording started when the mouse was able to run along the runway in a straight line without pauses. The measured step parameters were as follows: stride length (mean distance between each footprint) and stance width (mean distance between left and right footprints). For each paw of every mouse, 3–4 consecutive footprints were used for further calculations and comparisons.

### Statistics

Data were analyzed using STATISTICA 9.0 software (StatSoft, Krakow, Poland). Values are presented as means and standard deviation (SD). Mann–Whitney *U* test was conducted when simple independent pair-wise comparisons of variables were needed. For behavioral tests, test of analysis of variance (ANOVA) was used. Between-groups or between-groups with repeated measures comparisons were used when deemed appropriate. When significant interactions were present, follow-up analyses were performed by separate one- or two-way ANOVAs between variables. Differences were considered significant at *p* < 0.05.

## Results

### Body Weight, Ceruloplasmin Concentration and Liver Changes

There was no significant difference between body weight of young toxic milk and control mice. Old *txJ* mice had lower body mass than the control group (Table 1). No significant difference was noted between males and females within groups.

**Table 1:** Morphometric characteristics of mice used in the experiment and serum ceruloplasmin concentration

	Age (months)	Control mice	Toxic milk mice
Body weight (g), mean ± SD	2	21.8 ± 1.6	20.0 ± 2.1
	12	30.4 ± 1.8	25.1 ± 2.3 (*p* < 0.002)
Liver weight (g), mean ± SD	2	1.0 ± 0.1	1.1 ± 0.1
	12	1.4 ± 0.1	1.5 ± 0.2
Liver weight/body weight ratio, mean ± SD	2	0.05 ± 0.02	0.05 ± 0.01
	12	0.05 ± 0.003	0.05 ± 0.005
Spleen weight (g), mean ± SD	2	0.08 ± 0.01	0.07 ± 0.01
	12	0.1 ± 0.03	0.3 ± 0.02 (*p* < 0.003)
Spleen weight/body weight ratio, mean ± SD	2	0.004 ± 0.0001	0.004 ± 0.0001
	12	0.004 ± 0.0003	0.01 ± 0.005 (*p* < 0.0006)
Ceruloplasmin (mg/dl), mean ± SD	2	24.8 ± 3.1	7.2 ± 4.4 (*p* < 0.03)
	12	26.1 ± 2.0	4.5 ± 7.5 (*p* < 0.0003)

Each experimental group comprised 8–10 animals, *p* value is given for significant differences between genotypes in mice of the same age

The ceruloplasmin concentration in serum was lower in toxic milk mice than in the control group irrespective of the animals’ age (Table 1).

**Fig. 1:**
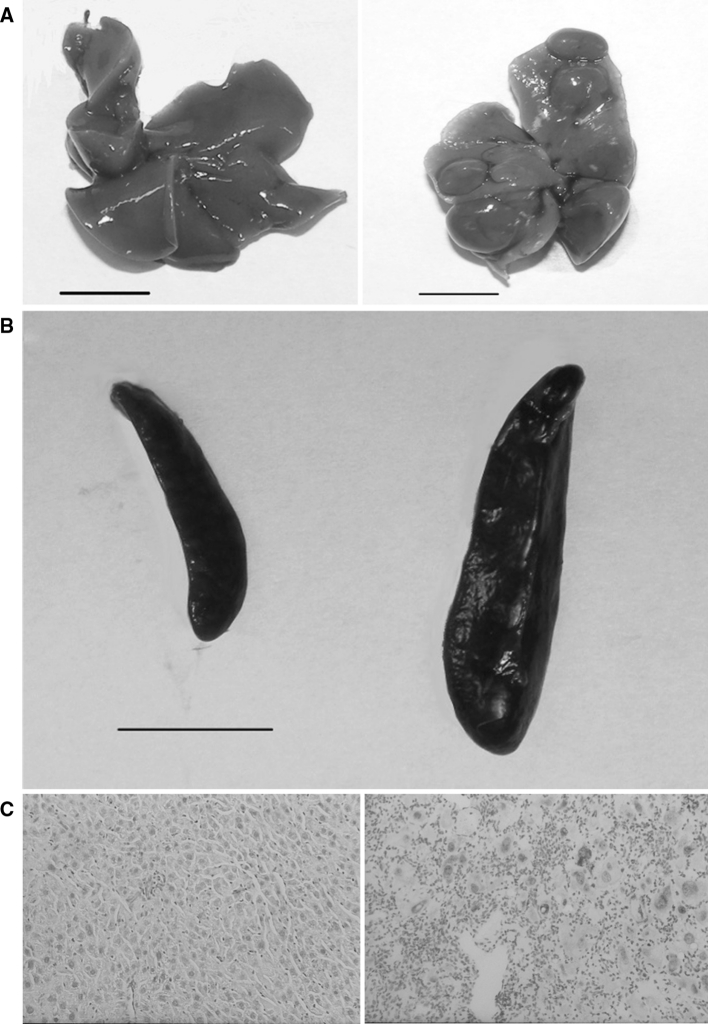
Macroscopic and microscopic changes in the organs of toxic milk mice. Control mouse liver (*left*) and toxic milk mouse liver (*right*) illustrating pathological changes at 12 months, *scale bars* 1 cm. **a** Normal spleen (*left*) of control mouse and enlarged spleen (*right*) of toxic milk mouse, *scale bar* 1 cm. **b** Unremarkable histology of the 12-month-old control mouse liver (*left*). Enlarged hepatocytes, inflammation, necrosis and copper deposits (*red*) in the liver of 12-month-old toxic milk mouse (*right*). Rhodanine with Mayer’s haematoxylin staining, original magnifications: ×200 (**c**) (Color figure online)

Macroscopically, livers of old toxic milk mice showed irregular surface, nodularity and pale color (Fig. 1a). Additionally, spleens of these mice were markedly enlarged due to passive congestion likely resulting from portal hypertension (Fig. 1b; Table 1). The microscopic morphology of the liver in young animals was normal and was similar in both experimental groups. The liver picture of 12-month-old *txJ* mice was remarkable, variability in the size of hepatocytes, necrosis and inflammation were observed (Fig. 1c). Moreover, deposits of copper with rhodanine stain were visualized.

### Metals Concentrations in Brain

**Fig. 2:**
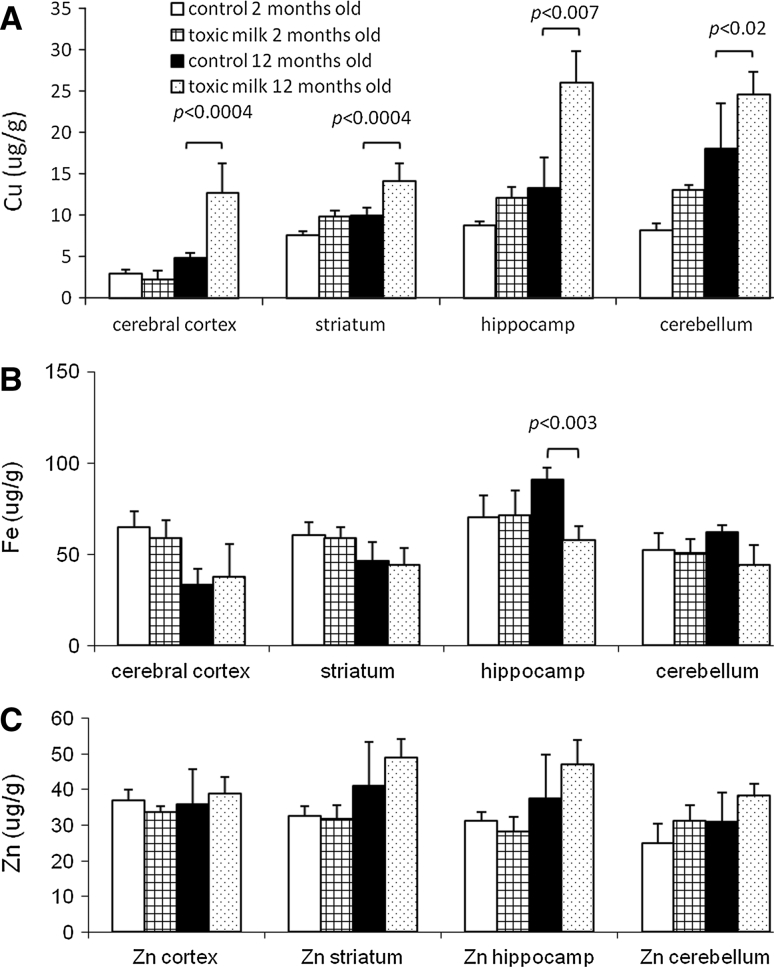
The toxic milk mice accumulate copper in different regions of brain at 12 months of age. Copper (**a**), iron (**b**) and zinc (**c**) in four brain regions of toxic milk and control mice. *Bars* indicate mean values ± SD, *n* = 8–10 animals/group, *p* value is given for significant differences between controls and toxic milk mice of the same age

Copper concentration in *txJ* mice brains was significantly increased at the age of 12 months. The old toxic milk mice had approximately twofold higher mean brain copper concentration than control mice (Fig. 2a). The increase in copper content was statistically significant in all brain regions examined.

No significant strain or age differences were detected in terms of iron content with the exception of hippocampus of old toxic milk mice, where iron concentration was markedly reduced in comparison to the control group (Fig. 2b). Brain zinc was higher in old *txJ* mice, but the difference was not statistically significant (Fig. 2c).

### Brain Monoamines and Tyrosine Hydroxylase, Dopamine Beta-Hydroxylase and Tryptophan Hydroxylase Expression

There were minor differences in concentration of DA and NA between toxic milk and controls in both age groups in various brain structures (Table 2). There was a slight reduction in dopamine content in the striata of old toxic milk mice. The serotonin content was significantly higher in 12-month-old toxic milk mice in comparison to control animals in all examined brain regions.

**Table 2:** Monoamine content in the various brain regions of toxic milk and control mice

Brain region	Age (months)	DA (pg/mg tissue), mean ± SD		NA (pg/mg tissue), mean ± SD		5-HT (pg/mg tissue), mean ± SD	
		Control	Toxic milk	Control	Toxic milk	Control	Toxic milk
Cerebral cortex	2	25.9 ± 33.2	65.5 ± 25.3	364.5 ± 43.2	376.7 ± 87.9	294.7 ± 39.9	342.6 ± 52.3
	12	201.7 ± 79.1	236.9 ± 85.9	315.4 ± 33.3	303.2 ± 46.7	401.3 ± 130.4	544.6 ± 127.5 (*p* < 0.02)
Striatum	2	11,465.9 ± 2,166.5	10,172.9 ± 1,352.7	69.6 ± 33.6	122.5 ± 51.6	423.1 ± 51.9	451.1 ± 37.3
	12	12,639.1 ± 1,820.9	10,171.8 ± 1,125.8 (*p* < 0.006)	81.3 ± 32.9	146.6 ± 37.6	290 ± 63.2	411 ± 110.9 (*p* < 0.007)
Hippocamp	2	18.5 ± 19.4	28.3 ± 36.9	285.1 ± 34.30	319.3 ± 92.8	208.9 ± 47.3	245.4 ± 26.1
	12	81.9 ± 39.9	83.9 ± 42.3	332.4 ± 58.0	349.2 ± 56.0	432.8 ± 103.1	598.2 ± 229.3 (*p* < 0.02)
Cerebellum	2	12.9 ± 20.7	18.51 ± 3.9	218.3 ± 40.6	176.1 ± 46.2	46.5 ± 40.7	65.5 ± 21.4
	12	31.55 ± 23.3	36.5 ± 22.4	164.1 ± 56.40	146.0 ± 47.2	145.6 ± 42.6	213.3 ± 75.4 (*p* < 0.04)

*n* = 8–10/group, *p* value is given for significant differences between genotypes in mice of the same age

**Fig. 3:**
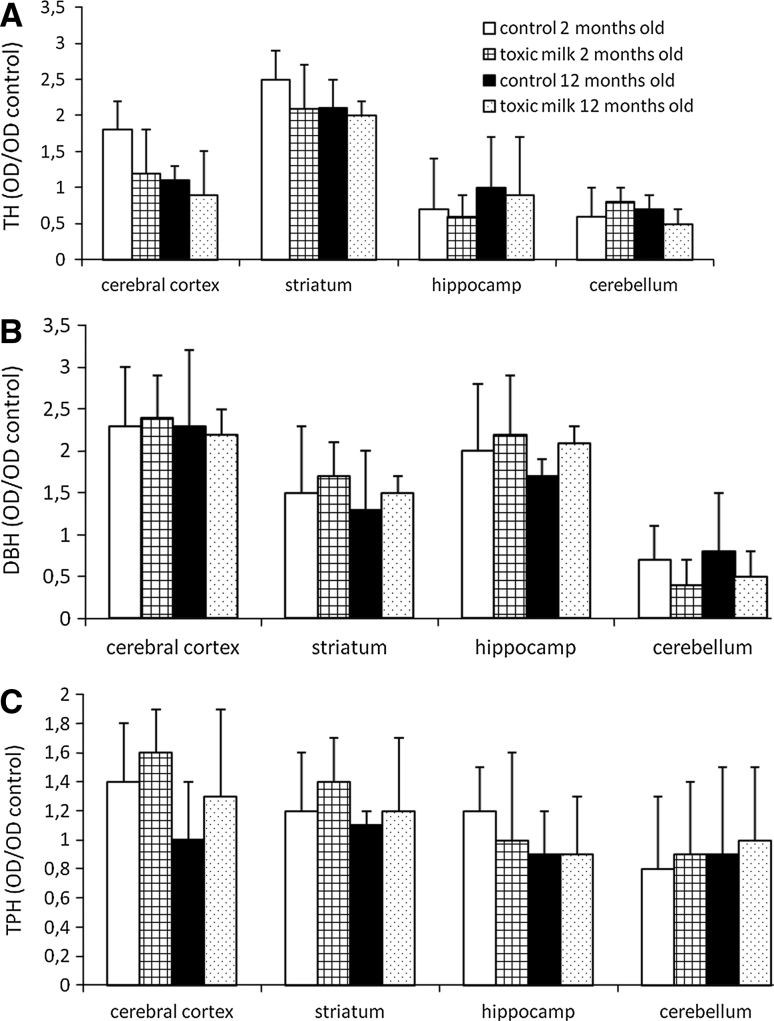
No evidence of neuronal loss as measured by expression of tyrosine hydroxylase (TH, **a**), dopamine beta-hydroxylase (DBH, **b**) and tryptophan hydroxylase (TPH, **c**) in brains of toxic milk mice and controls. *Bars* indicate mean values ± SD of optical density (OD) of examined sample in relation to optical density of control sample, *n* = 8–10 animals/group

No significant changes in the expression of TH, DBH or TPH were noted between toxic milk mice and control groups in all the examined brain structures and both age groups (Fig. 3a–c).

### Behavioral Tests

**Fig. 4:**
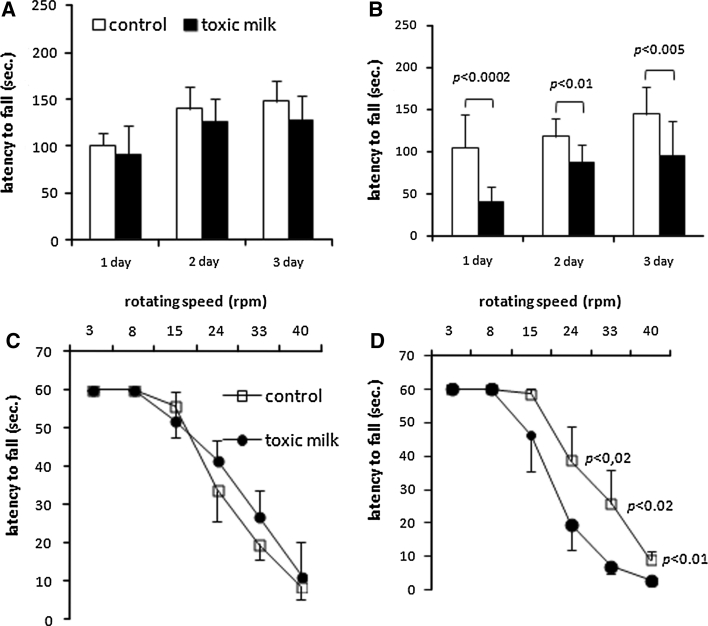
Accelerating rotarod and fixed-speed rotarod identification of locomotor deficits in 12-month-old toxic milk mice. Latency to fall off the rotarod of toxic milk mice (*black symbols* or *black bars*) and control mice (*white symbols* or *white bars*). Accelerating rotarod protocol results of 2- (**a**) and 12-month-old mice (**b**). Fixed speed rotarod exercise results of 2- (**c**) and 12-month-old mice (**d**). *Symbols* and *bars* indicate mean values ± SD, *n* = 8–10 animals/group, *p* value is given for significant differences between mice of the same age

On the standard accelerating rotarod, 12-month-old toxic milk mice fell off significantly earlier than control mice [genotype, *F*_(1,15)=_ 16.5; *p* < 0.0004, Fig. 4b]. The difference was significant through the 3-day trial period [day 1: *F*_(1,15)_ = 20.7; *p* < 0.0002; day 2: *F*_(1,17)_ = 6.8; *p* < 0.01; *F*_(1,15)_ = 9.3; *p* < 0.005; Fig. 4b].

When tested on the fixed-speed rotarod, 2- and 12-month-old toxic milk and control mice performed equally well at speeds up to 15 rpm [genotype, *F*_(2,11)_ = 2.76; *p* < 0.1, Fig. 4c, d]. The latency to fall of 12-month-old toxic milk mice at the higher rotation speeds of 24–40 rpm [genotype, *F*_(2,11)_ = 4.6; *p* < 0.02] was significantly shorter in comparison to control group (Fig. 4d).

**Fig. 5:**
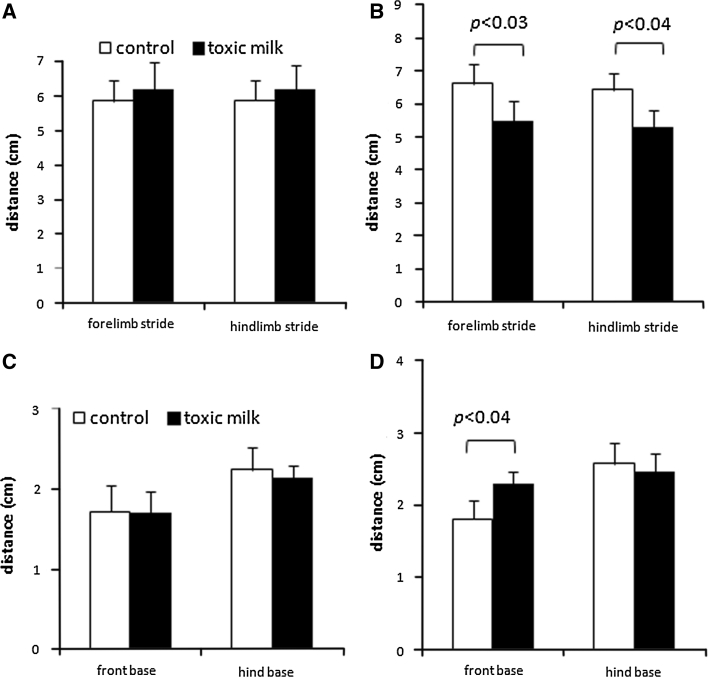
Footprints tests revealing major gait abnormalities in 12-month-old toxic milk mice. Stride lengths of toxic milk mice (*black bars*), compared to that of control mice (*white bars*) of various ages: 2- (**a**), and 12-month-old (**b**). Stance width of 2- (**c**) and 12-month-old mice (**d**). *Bars* indicate mean values ± SD, *n* = 8–10 animals/group, *p* value is given for significant differences between toxic milk mice and control mice of the same age

Quantitative analysis of footprints from 12-month-old mice showed significant abnormalities in *txJ* mice compared to controls. *txJ* mice had a shorter stride length [fore paws *F*_(1,11)_ = 3.6; *p* < 0.03; hind paws *F*_(1,11)_ = 3.1; *p* < 0.04; Fig. 5b]. The stance width of the hind paws in 12-month-old toxic milk mice did not differ from that of their C3HeB/FeJ littermates [genotype, *F*_(1,11)_ = 0.9; *p* < 0.4; Fig. 5d], while that of the fore paws was significantly broader in toxic milk mice compared with control mice [genotype, *F*_(1,11)_ = 5.0; *p* < 0.04].

## Discussion

In this study, impaired locomotor performance of aged toxic milk mice accompanied by increase in copper and serotonin content in different brain regions and slight decrease in dopamine concentration in the striatum was demonstrated.

The gait disturbances and impaired motor coordination in 12-month-old *txJ* mice resemble neurological symptoms described in patients with WND. The typical pathology observed in humans encompasses bilateral lesions in the putamen, globus pallidus, caudate nucleus and other brain regions [9]. The reason for such specific localization of neuronal damage in WND is not yet fully understood. It is anticipated that vulnerability of neurons to copper overload may be region dependent because substantia nigra, tail of caudate nucleus and putamen are the brain structures characterized by the highest copper and iron concentration in humans [9, 15, 16]. The local distribution of copper and iron in brains of toxic milk mice is similar to that in humans [7, 17]. However, expression analysis of enzymes specific for noradrenergic, serotoninergic or dopaminergic neurons in various brain regions failed to prove excessive destruction of neurons in *txJ* mice. Observations from the present study are in concordance with results of other studies on neuronal injury in animal models of WND [7, 18]. Thus, it can be stated that despite similar symptoms, neuropathology triggered by genetic ATP7B dysfunction is different in humans and animals.

There are limited reports on neurotransmitters in WND. The concentration of HVA, 5HIAA and MHPG in cerebrospinal fluid of WND individuals is low, reflecting the generalized loss of white and grey matter [10, 11]. There is a marked loss of striatal dopamine transporters in patients with neurological presentation of the disease [19]. Moreover, impaired glucose consumption has been described in dopaminergic brain regions of WND patients [20]. The decrease in DA content in striatum in 12-month-old *txJ* mice is in concordance with observations in WND mentioned above [19, 20].

There are a few reports on catecholamines in animal models of WND. In young (11 weeks) LEC rats, high DA and low NA content in striatum have been reported, which probably reflects compromised activity of cuproenzyme DBH [21]. The young LEC rats present copper deficiency in brain, while in old animals the copper concentration in brain is increased [22]. In another study the density of TH-immunoreactive fibers was significantly lower in various brain regions in comparison to controls in animals aged 4 and 10 weeks [23]. The differences in striatal concentration of DA and NA as well as in the density of TH-positive fibers disappear in older LEC rats (20 weeks) as the copper load in the brain increases. Conversely, 5-HT brain content as well serotoninergic fiber density continuously increases with age of the animals [23].

In the experiment, 5-HT concentration was slightly increased in the brains of young mutant mice in comparison to controls and increased evidently in aged animals. There are at least two hypothetical explanations for this observation. Firstly, toxic milk mice and LEC rats independent of age are characterized by reduced ceruloplasmin activity as assessed by enzymatic assays. Ceruloplasmin was proven to oxidize serotonin in vitro, thus high serotonin concentration may result from impaired metabolism; however, ceruloplasmin-dependent oxidation of serotonin has not been observed in vivo [24, 25].

Pineal night-specific ATPase (PINA) is another hypothetical connection between *Atp7B* mutation and serotonin release. PINA is generated from alternative splicing of the *ATP7B* gene [26]. PINA expression exhibits a diurnal rhythm in both pineal gland and retina with greater expression at night than during the day. Additionally, 5-HT and melatonin synthesis displays a circadian rhythm in nocturnal rodents [27]. The 5-HT content and secretion increases when melatonin formation is blocked [28, 29]. LEC rats lack PINA in the pineal gland [30]. Thus far, there is no published information regarding PINA production in toxic milk mice. If PINA is involved in the regulation of melatonin synthesis, impaired PINA formation could be responsible for excessive release of serotonin in *txJ* mice and LEC rats.

In human with WND, some gender-associated differences have been reported [31, 32]. In this study, no significant sex-related differences were observed in results of behavioral or neurochemical tests in toxic milk mice; however, these observations are preliminary and should be confirmed and verified in dedicated studies involving a larger numbers of same-sex animals.

It was observed that behavioral changes in toxic milk mice can result from copper-induced neuronal injury but there may be other probable causes as well. Aged toxic milk mice suffer from liver disease, so worse locomotor performance of toxic milk mice may reflect their generally poor physical condition. Inflammatory response to copper overload in the brains of toxic milk mice has been documented, which can contribute to worse cognitive and motor skills [7]. Finally, it cannot be excluded that impaired liver function in toxic milk mice is linked to augmented production of toxic metabolites that disturb brain function as it is observed in humans with liver failure. Altogether these uncertainties need further investigations.

Results from the present study confirm that the phenotype of toxic milk mice resembles clinical symptoms of WND. However, brain lesions in 12-month-old *txJ* mice are not as advanced as in patients with the neurological form of WND. Further exploration of neuronal injury in toxic milk mice is warranted which may contribute to a better understanding of WND neuropathology.

## Abbreviations

WND: Wilson’s disease
LEC: Long Evans Cinnamon
*txR*: Toxic milk mice strain described by H. Rauch
*txJ*: The Jackson Laboratory toxic milk mice
